# Secondhand smoke exposure among United States children with functional disabilities: National Health and Nutrition Examination Survey, 2021–2023

**DOI:** 10.1016/j.pmedr.2025.103183

**Published:** 2025-07-22

**Authors:** Raed A. Bahelah

**Affiliations:** Department of Population Health Science, John D. Bower School of Population Health, University of Mississippi Medical Center, Jackson, MS, USA

**Keywords:** Secondhand smoke, Passive smoking, Disability, Children, NHANES

## Abstract

**Objective:**

Secondhand smoke exposure (SHSe) poses significant health problems. This study aims to examine the prevalence and factors associated with SHSe among 5–17 years old U.S. children with functional disabilities.

**Methods:**

NHANES 2021–2023 applied Child Functioning Module to assess functional disabilities among 5–17 years old U.S. children. SHSe was defined as living in the same household with a person who is a tobacco smoker.

**Results:**

Over three million (32.9 %) children with functional disabilities were exposed to SHS. Children with functional disabilities had higher odds of SHSe compared with children without functional disabilities (Adjusted Odds Ratio “AOR” =1.79, 95 % CI = 1.45, 2.23). Among children with functional disabilities, Hispanic children had lower odds of SHSe compared with non-Hispanic White children (AOR = 0.36, 95 % CI = 0.18, 0.72). The odds of SHSe among children with functional disabilities were negatively associated with the household reference person's educational level (less than high school: AOR = 11.86, 95 % CI = 3.26, 43.16; high school/general educational development/some college: AOR = 6.36, 95 % CI = 2.53, 15.98; ≥ college degree as the reference).

**Conclusions:**

Disparities in SHSe at home by education level and race/ethnicity among U.S. children with functional disabilities are noted and warrant tailored interventions to reduce SHSe.

## 1 Introduction

Secondhand smoke exposure (SHSe) is a public health problem that exposes non-smokers to significant adverse health outcomes, including cardiac and cerebrovascular diseases, and lung cancer (Centers for Disease Control and Prevention, 2024a). Although the prevalence of SHSe in the United States (U.S.) has declined, disparities in SHSe persisted (Shastri et al., 2021). It has been reported that adults with disabilities are more likely to be exposed to SHS than those without disabilities (Hall et al., 2013).

Children exposed to SHS are at a higher risk of sudden infant death syndrome, respiratory and ear infections, and repeated asthmatic attacks (Centers for Disease Control and Prevention, 2024a). SHSe is associated with negative childhood neurobehavioral outcomes such as learning disabilities, attention-deficit/hyperactivity disorder, and behavioral disorders (Kabir et al., 2011). Younger children are more likely to be exposed to SHS than older children which is concerning given the tremendous impact on a growing body and neurobehavioral developments among very young children (Shastri et al., 2021). There is a gap in our knowledge concerning the prevalence of SHSe among children with functional disabilities and what factors are associated with SHSe. The current study aims to compare the prevalence of SHSe at home (hereafter, SHSe) between children with and without functional disabilities, and to examine the factors associated with SHSe among 5–17 years-old U.S. children with functional disabilities.

## 2 Materials and methods

### 2.1 Data source

Data come from the most recent cycle of the National Health and Nutrition Examination Survey (NHANES) 08/2021–08/2023. NHANES is a national survey of the US population to collect information about health and nutritional status (Centers for Disease Control and Prevention, 2022). Since 1999, NHANES has continuously recruited a representative sample of the civilian, non-institutionalized U.S. population of all ages, and the survey data are released every two years. NHANES uses a complex, multi-stage sampling design with household interviews and examinations of a subsample of participants at a Mobile Examination Center (Centers for Disease Control and Prevention, 2022). NHANES 08/2021–08/2023 is the first data collected after the COVID-19 pandemic, which demanded changes in sampling methodology and data collection. Additional weighting adjustments to account for declining response rates and alteration of sampling methods at the household level were undertaken to minimize bias.

Starting in the 2019–2020 cycle, NHANES administered questions to assess functioning among adults and children. However, because the 2019–2020 cycle included data from a convenience sample due to the interruption of the data collection during the COVID-19 pandemic, it was not feasible to combine data from the 2019–2020 and the 2021–2023 cycles. Therefore, the current study uses data on functioning from the 2021–2023 cycle only. The current study uses de-identified and publicly available NHANES data, determined to be exempt from review by the University of Mississippi Medical Center's Institutional Review Board.

### 2.2 Measures

#### 2.2.1 Outcome variable

The number of people who live in the household and smoke was evaluated using the survey question: “How many people who live here smoke cigarettes, cigars, little cigars, pipes, water pipes, hookah, or any other tobacco product?”. This question has the following possible responses: none in the household is a smoker, one household member is a smoker, ≥ two household members are smokers, refused, or don't know. SHSe was categorized as yes (at least one household member is a smoker) or no (none in the household is a smoker). Responses “Refused” and “don't know” were removed from the analysis.

#### 2.2.2 Covariates

NHANES administered 16 questions from the Child Functioning Module (CFM) to assess functional disabilities among children aged 5–17 years. CFM was developed, tested, and validated by the Washington Group on Disability Statistics and the United Nations Children's Fund with the purpose of providing accurate population-based estimates of children with functional disabilities (Washington Group on Disability Statistics, 2025). NHANES provided a disability indicator (Yes/No) based on evaluation of several areas of difficulty, including difficulty with seeing, hearing, use of equipment for walking, difficulty with self-care, concentration, and remembering, among others. Specifically, those with ‘a lot of difficulty’ or ‘cannot do at all’ any of the following functions: seeing, hearing, mobility, self-care, communication, learning, remembering, concentrating, accepting change, controlling behavior, and making friends, or reported feeling anxious or sad/depressed everyday, were classified as having functional disabilities (Centers for Disease Control and Prevention, 2022).

Child's race (White, Black, Hispanic, Other), age in years (5–8, 9–12, 13–17), and sex (male, female) were examined. Household reference person's sex (male, female), age in years (<40, ≥40), and educational level (less than high school, high school graduate/general educational development “GED”/some college, college graduate or above) were included. A reference person is defined as the first adult (≥18 years old) household member listed on the household member roster who owns or rents the household residence. Household reference data are often used in NHANES as socioeconomic characteristics for youth (Centers for Disease Control and Prevention, 2022). The ratio of family income to poverty (a lower ratio indicates higher poverty) was also included.

### 2.3 Statistical analysis

**Fig. 1:**
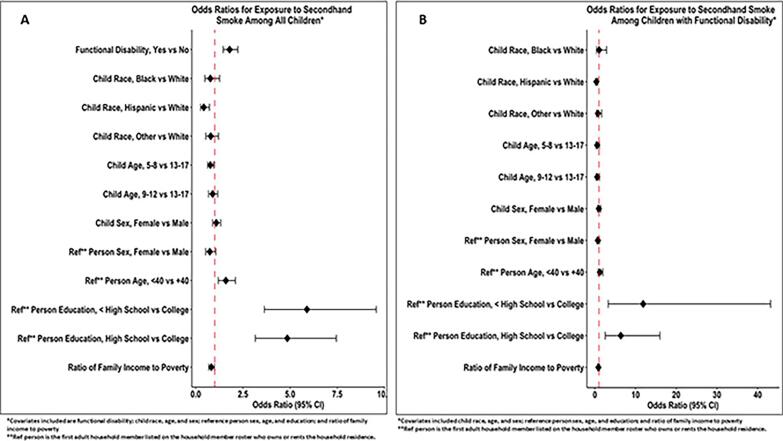
Adjusted Odds Ratios for the Exposure to Secondhand Smoke among 5–17 Years Old United States Children: National Health and Nutrition Examination Survey, 2021–2023. Panel A includes all children, and Panel B includes children with functional disability.

Children with complete data on functional disabilities were included (*N* = 2786). After excluding children who smoked tobacco in the past five days (using NHANES variable “SMQ681”, n = eight), the final analytic sample was *N* = 2778. Weighted prevalence estimates of functional disabilities by sample characteristics were calculated and accounted for NHANES's complex survey design (Table 1). Rao-Scott Chi-Square test was applied to test the bivariable association between categorical variables, and the Mann-Whitney *U* test for the difference between medians. Two weighted logistic regression models were performed. The first model compared the odds of SHSe by functional disabilities among all children (Fig. 1-A), while the second model examined the factors associated with SHSe among children with functional disabilities (Fig. 1-B). Both models controlled for all covariates simultaneously. Model fit was evaluated using the Hosmer-Lemeshow test (Hosmer et al., 2013). The function “svyglm” from the “survey” package was used for the logistic regression modeling, while the function “svyCreateTableOne” from “tableone” package was used to calculate Rao-Scott Chi-Square and Mann-Whitney *U* tests. All analyses were two-tailed, with *p* < 0.05 considered statistically significant. The R software was used for all analyses.

**Table 1:** Baseline characteristics of 5–17 Years Old United States children by exposure to secondhand smoke, national health and nutrition examination survey, 2021–2023.

Characteristic	Exposure to Secondhand Smoke				*p*-value⁎
	No (weighted prevalence = 37,166,945)		Yes (weighted prevalence = 11,260,450)		
	Frequency (%)	Weighted prevalence (%)	Frequency (%)	Weighted prevalence (%)	
Sample: all children (with and without functional disabilities)					
Functional Disability No Yes	1517 (76.8) 308 (63.9)	31,034,631 (79.0) 6,132,313 (67.0)	457 (23.2) 174 (36.1)	8,246,456 (21.0) 3,013,991 (33.0)	<0.01
Race/Ethnicity (Child) White Black Hispanic Other	732 (74.9) 253 (69.1) 550 (77.2) 290 (72.3)	17,275,724 (77.7) 4,088,868 (71.5) 9,834,719 (78.8) 5,967,633 (74.6)	245 (25.1) 113 (30.9) 162 (22.8) 111 (27.7)	4,956,610 (22.3) 1,630,214 (28.5) 2,645,765 (21.2) 2,027,857 (25.4)	0.49
Sex (Child) Male Female	945 (75.7) 880 (72.9)	19,698,280 (77.6) 17,468,665 (75.9)	304 (24.3) 327 (27.1)	5,699,316 (22.4) 5,561,131 (24.1)	0.30
Age in years (Child) 5–8 9–12 13–17	586 (74.6) 582 (75.2) 657 (73.3)	11,185,523 (77.8) 11,423,629 (77.3) 14,557,793 (75.6)	200 (25.4) 192 (24.8) 239 (26.7)	3,198,560 (22.2) 3,359,593 (22.7) 4,702,294 (24.4)	0.55
Household Reference Person Sex Male Female	775 (74.0) 1047 (74.6)	16,542,856 (76.3) 20,589,246 (77.2)	273 (26.0) 356 (25.4)	5,143,311 (23.7) 6,080,621 (22.8)	0.77
Household Reference Person Age in years <40 ≥40	707 (66.8) 1118 (79.9)	13,143,515 (69.5) 24,023,430 (81.4)	351 (33.2) 280 (20.1)	5,770,282 (30.5) 5,490,166 (18.6)	<0.01
Household Reference Person Education Level < High School High School/GED⁎⁎/Some College ≥ College	221 (63.0) 854 (66.3) 703 (93.1)	3,837,843 (63.8) 16,116,328 (67.9) 16,224,691 (93.3)	130 (37.0) 434 (33.7) 52 (6.9)	2,173,607 (36.2) 7,602,775 (32.1) 1,169,318 (6.7)	<0.01
Ratio of family income to poverty (median (IQR))	–	2.77 (1.44, 4.59)	–	1.46 (0.77, 2.76)	<0.01

Sample: children with functional disabilities					
Race/Ethnicity (Child) White Black Hispanic Other	137 (61.7) 33 (61.1) 98 (70.0) 40 (60.6)	3,080,004 (66.4) 471,118 (56.2) 1,763,607 (71.7) 817,584 (67.6)	85 (38.3) 21 (38.9) 42 (30.0) 26 (39.4)	1,558,938 (33.6) 367,799 (43.8) 695,233 (28.3) 392,021 (32.4)	0.52
Sex (Child) Male Female	170 (66.4) 138 (61.1)	3,365,958 (68.5) 2,766,355 (65.4)	86 (33.6) 88 (38.9)	1,550,326 (31.5) 1,463,665 (34.6)	0.52
Age in years (Child) 5–8 9–12 13–17	87 (64.9) 93 (67.4) 128 (61.0)	1,733,474 (70.3) 1,799,176 (70.3) 2,599,663 (63.1)	47 (35.1) 45 (32.6) 82 (39.0)	732,622 (29.7) 758,939 (29.7) 1,522,429 (36.9)	0.45
Household Reference Person Sex Male Female	122 (63.2) 185 (64.2)	2,593,866 (65.5) 3,523,832 (68.1)	71 (36.8) 103 (35.8)	1,364,939 (34.5) 1,649,051 (31.9)	0.59
Household Reference Person Age in years <40 ≥40	123 (56.2) 185 (70.3)	2,268,710 (60.0) 3,863,604 (72.0)	96 (43.8) 78 (29.7)	1,512,192 (40.0) 1,501,799 (28.0)	0.01
Household Reference Person Education Level < High School High School/GED⁎⁎/Some College ≥ College	28 (43.8) 168 (56.9) 104 (92.0)	482,043 (24.2) 3,087,099 (58.5) 2,368,321 (90.5)	36 (56.3) 127 (43.1) 9 (8.0)	1,512,192 (75.8) 2,186,512 (41.5) 247,166 (9.5)	<0.01
Ratio of family income to poverty (median (IQR))	–	2.34 (1.29, 4.18)	–	1.25 (0.73, 2.61)	<0.01

⁎ p-value is based on Rao-Scott chi-square test or Mann-Whitney *U* test.

⁎⁎ GED = General Educational Development.

## 3 Results

In 2021–2023, over 9 million (18.9 %) U.S. children had functional disabilities, and over 11 million (23.3 %) lived in a residence where at least one household member is a tobacco smoker (i.e., exposed to SHS). More than three million (32.9 %) children with functional disabilities were exposed to SHS. Table 1 summarizes the characteristics of SHSe by functional disabilities, child, and household reference person characteristics. Children with functional disabilities had higher SHSe than children without functional disabilities (32.9 % vs 21 %, *p* < 0.01). Among all children, those living with a younger household reference person (<40 years old) have higher SHSe (30.5 %) compared with those living with a household reference person ≥40 (18.6 %) (p < 0.01). SHSe decreased with increasing educational level of the household reference person (36.2 % for <high school, 32.1 % for high school/GED/some college, and 6.7 % for ≥ college degree, p < 0.01). A higher median ratio of family income to poverty was associated with lower SHSe (p < 0.01). Similar findings were observed in the sample limited to children with functional disabilities (Table 1).

Fig. 1 shows the adjusted odds ratios (AOR) for SHSe by functional disabilities in the full sample (Panel A) and among children with functional disabilities (Panel B). Children with functional disabilities had higher odds of SHSe compared with children without functional disabilities (AOR = 1.79, 95 % CI = 1.45, 2.23) (Fig. 1-A). Compared with White children with functional disabilities, Hispanic children with functional disabilities had lower odds of SHSe (AOR = 0.36, 95 % CI = 0.18, 0.72). The odds of SHSe among children with functional disabilities was significantly higher when the reference person in the household has less than high school degree (AOR = 11.86, 95 % CI = 3.26, 43.16) or high school/GED/some college degree (AOR = 6.36, 95 % CI = 2.53, 15.98), compared to the reference category where the reference person has a college degree or higher (Fig. 1-B). Hosmer-Lemeshow test showed an acceptable fit for both models.

## 4 Discussion

To the best of my knowledge, this is the first study to examine the prevalence and factors associated with SHSe among U.S. children with functional disabilities. Using a validated tool for the assessment of functional disabilities among 5–17 years old children, the current study found that over three million (32.9 %) U.S. children with functional disabilities were exposed to SHS at home. This study also unveiled racial/ethnic disparities where Hispanic children with functional disabilities were less likely to be exposed to SHS compared to White children with functional disabilities. Additionally, children with functional disabilities were less likely to be exposed to SHS at home if the household reference person holds a college degree or higher. These findings show the pressing need for public health efforts to reduce SHSe in the home among all children, and children with functional disabilities in particular, because of a higher exposure and potentially greater risk of complications associated with SHSe compared with children without functional disabilities.

Although SHSe among U.S. children has declined (Merianos et al., 2019), disparities still exist. Compared with non-Hispanic White children, non-Hispanic Black children were more likely to be exposed to SHS, while non-Hispanic Other, Hispanic, and Mexican children were less likely to be exposed to SHS (Merianos et al., 2019). The current study found that Hispanic children were less likely to be exposed to SHS, compared with White children. Poverty is strongly associated with SHSe among children (Merianos et al., 2019; Shastri et al., 2021). The current study found that children with functional disabilities from households where the reference person holds a college degree or higher had lower odds of SHSe. Education is a proxy of income, but can also influence healthy behaviors by making informed decisions. Disparities in the enforcement of smoke-free policies in homes in the U.S. are known (Mantey et al., 2022) and can possibly be explained by the child's race and the education level of the household reference person, as shown in the current study. The novelty of the current study stems from its focus on children with functional disabilities and adds to the literature that racial/ethnic and educational disparities in SHSe extend to children with functional disabilities.

Strengths of this study include the use of a national dataset and the assessment of functional disability based on a validated tool. The presence of smokers in the home is an accurate tool for the assessment of SHSe among children (Ksinan et al., 2021). Limitations are noted. SHSe was not verified using biochemical tests. Unfortunately, data on cotinine measurements were not available in the NHANES 2021–2023 cycle. Even if smoking household members did not smoke inside homes, smoke can travel through doors and windows and expose household members who are not smokers to SHS (Centers for Disease Control and Prevention, 2024b). The cross-sectional nature of the study allows the examination of associations, but not causation, among the studied variables. Children who smoked any tobacco in the past five days were excluded, but it is possible that tobacco was smoked before five days. Lastly, housing type (renting, owning) can be associated with SHSe (Merianos et al., 2019), but this variable was not available from the NHANES 2021–2023 cycle.

In conclusion, a significant number of U.S. children with functional disabilities are exposed to SHS, especially White non-Hispanic children, while a higher educational level of the reference person in the household was associated with lower exposure. These findings call for the need to raise awareness of the dangers of SHSe and for the protection of all children, including children with functional disabilities, from the harmful effects of SHSe at home and in other places.

## CRediT authorship contribution statement

**Raed A. Bahelah:** Writing – review & editing, Writing – original draft, Validation, Methodology, Formal analysis, Data curation, Conceptualization.

## Declaration of competing interest

The authors declare that they have no known competing financial interests or personal relationships that could have appeared to influence the work reported in this paper.
